# Somatic intronic *TP53* c.375+5G mutations are a recurrent but under‐recognized mode of *TP53* inactivation

**DOI:** 10.1002/cjp2.242

**Published:** 2021-09-10

**Authors:** M Herman Chui, Ciyu Yang, Nikita Mehta, Vikas Rai, Ahmet Zehir, Amir Momeni Boroujeni, Marc Ladanyi, Diana Mandelker

**Affiliations:** ^1^ Department of Pathology Memorial Sloan Kettering Cancer Center New York NY USA

**Keywords:** p53, *TP53*, intronic mutation, pathogenic variant, cancer

## Abstract

*TP53* is one of the most ubiquitously altered genes in human cancer. The biological impact of rare variants, particularly those located within noncoding regions, remains poorly understood. From interrogation of clinical massively parallel sequencing data from over 55,000 tumors, which included 23,330 tumors with known *TP53* mutations, *TP53* intron 4 nucleotide substitutions at position c.375+5G were identified in 45 tumors (0.2% of *TP53*‐mutated cancers), comprising cancers of different organ sites. Loss‐of‐heterozygosity or a second‐hit somatic *TP53* mutation was observed in 34 of 40 (85%) informative cases. RT‐PCR analysis showed the c.375+5G>T variant to be associated with aberrantly spliced *TP53* mRNA transcripts with concomitant loss of the normal transcript. Immunohistochemical staining for p53 was performed on a representative subset of tumors with *TP53* c.375+5G variants (*n* = 14), all of which showed loss of protein expression (100%; *n* = 13 complete loss, *n* = 1 subclonal loss). Our data are consistent with classification of *TP53* c.375+5G variants as deleterious intronic mutations that interfere with proper mRNA splicing, ultimately resulting in loss of expression of functional p53 protein. The clinical scenario of a tumor with loss of p53 immunohistochemical staining, yet lacking a detectable *TP53* exonic mutation, should therefore prompt consideration of splice‐altering intronic variants.

## Introduction

The *TP53* tumor suppressor gene is the most frequently somatically altered gene in human cancer [1]. The majority of mutations occur in the DNA‐binding domain, resulting in loss of transcription factor function [1, 2]. Most of these are missense mutations, clustered at a few hot‐spots, although nonsense and frameshift mutations, causing truncation of the protein, constitute approximately 20–25% of mutations.

Immunohistochemical staining for p53 protein expression serves as a useful surrogate marker for mutation status [3, 4]. The pattern of immunohistochemical staining correlates with the type of *TP53* mutation. Missense mutations render the protein resistant to proteolytic degradation, resulting in nuclear accumulation of protein, manifested as diffuse immunohistochemical staining throughout the tumor. In contrast, truncating mutations result in loss of protein expression and complete absence of staining.

The advent of massively parallel sequencing has led to increased recognition of variants in noncoding regions. Mutations at canonical splice sites (i.e. conserved donor and acceptor sites) at the exon–intron boundaries result in aberrant splicing and are estimated to represent 2% of *TP53* mutations [2]. However, the effects of nucleotide variants at other positions on splicing and protein expression remain poorly understood, and tumor sequencing assays generally only report coding and canonical splice site mutations, omitting these deeper intronic variants from clinical reports.

The prototype of a *TP53* mutation‐driven cancer is high‐grade serous ovarian carcinoma, which has the highest prevalence of somatic *TP53* genetic alterations across human cancer types [1]. With mutational prevalence reported to be 95–100% across various studies [5, 6], *TP53* mutation is considered by many to be an essential diagnostic feature of this tumor subtype [7]. We recently conducted a retrospective analysis of our institutional cohort of high‐grade serous ovarian cancer patients subjected to tumor mutational profiling using the Memorial Sloan Kettering ‐ Integrated Mutation Profiling of Actionable Cancer Targets (MSK‐IMPACT) platform to identify rare cases that lack *TP53* genetic alterations [6]. Through this effort, we observed a recurrent intronic variant, c. 375+5G>T, in several tumors which did not harbor any other *TP53* sequence alteration. As the biological significance of this variant was unknown, the original tumor sequencing report had documented these cases as negative for *TP53* somatic genetic alterations. To explore the possibility that intronic nucleotide substitutions at position c.375+5G represent an alternative mechanism for disruption of the *TP53* gene, in this study we performed a comprehensive interrogation of these variants across a pan‐cancer cohort, complemented by mRNA and immunohistochemical analyses.

## Materials and methods

### Case selection and massively parallel sequencing data analysis

We performed a retrospective search of tumor samples, across all cancer types, from 55,772 patients subjected to clinical targeted massively parallel sequencing using the MSK‐IMPACT platform, to determine the prevalence of somatic *TP53* c. 375+5G variants. Specific details of panel design, capture protocol, sequencing, quality control, read alignment, and variant calling for the MSK‐IMPACT assay have been described [8]. Sequencing data from tumors harboring these variant alleles were analyzed for coexisting *TP53* exonic mutations. Fraction and Allele‐Specific Copy Number Estimates from Tumor Sequencing (FACETS) was used to infer copy number alterations and loss‐of‐heterozygosity (LOH) [9].

### *TP53* mRNA transcript analysis

RNA extraction and reverse transcription were performed on formalin‐fixed paraffin‐embedded (FFPE) tumor and adjacent normal tissue obtained from the surgical resection specimen of a high‐grade serous ovarian carcinoma with a *TP53* c. 375+5G>T variant. RT‐PCR was performed on cDNA from matched patient tumor and normal samples, and from peripheral blood mononuclear cells pooled from healthy individuals, followed by capillary electrophoresis and Sanger sequencing. To assess for aberrant splicing of intron 4, the following M13‐tagged primers were used: exon 3‐F: GTAAAACGACGGCCAGTCAGACCTATGGAAACTACTTCCTG; exon 4‐F: GTAAAACGACGGCCAGTCAATGGATGATTTGATGCTG; and exon 5‐R: CAGGAAACAGCT ATGACGGCAAAACATCTTGTTGAGG.

### Immunohistochemistry

Immunohistochemical staining for p53 was performed on FFPE tissue sections with the monoclonal antibody clone DO‐7 (Ventana Medical Systems, Tucson, AZ, USA; retrieval: CC1 32 min; incubation: 16 min; ready to use dilution) with external tissue controls (including tumors with known *TP53* mutations) for each run. Assessment of p53 immunostaining was performed by a pathologist (MHC) and classified as wild‐type (heterogeneous staining) or aberrant (diffuse or complete absence of staining) expression pattern.

## Results and discussion

Interrogation of our institutional database of 55,772 tumors from patients subjected to clinical somatic mutational profiling revealed 45 cases (0.08%) with nucleotide substitutions in intron 4 of *TP53* at position c. 375+5G (G>T, *n* = 23; G>A, *n* = 15; and G>C, *n* = 7). These were distributed over cancer types across organ sites, including those with a high frequency of *TP53* genetic alterations, such as ovarian high‐grade serous, colorectal, pancreatic, gastroesophageal, and lung carcinomas. For each tumor type, the prevalence was <1% (Table 1). The prevalence of known *TP53* mutations across all cancer types in our institutional cohort was 42% (*n* = 23,330), and thus c.375+5G somatic variants represent approximately 0.2% of all *TP53* mutations. Germline testing performed on 23,111 cancer patients at our institution revealed 53 patients with pathogenic *TP53* germline variants, and none with a c.375+5G germline variant.

**Table 1:** Distribution of *TP53* c.375+5G intronic variants across tumor types.

	*TP53* c.375+5G substitution (*n*)			Frequency (%)
	G>T	G>A	G>C	
Breast carcinoma	1	1	3	5/5,887 (0.08%)
Colorectal adenocarcinoma	3	2	2	7/4,859 (0.14%)
Esophageal/gastroesophageal junction adenocarcinoma	1	1	1	3/943 (0.32%)
Pancreatic adenocarcinoma	3	2	0	5/2,520 (0.20%)
Anal squamous cell carcinoma	1	0	0	1/126 (0.79%)
Gliomas^*^	2	4	0	6/2,037 (0.29%)
Lung carcinomas^†^	5	2	0	7/7,488 (0.09%)
Ovarian high‐grade serous carcinoma	5	0	1	6/1,441 (0.42%)
Uterine leiomyosarcoma	1	0	0	1/191 (0.52%)
Urothelial carcinoma	0	2	0	2/1,910 (0.10%)
Merkel cell carcinoma	0	1	0	1/138 (0.72%)
Cancer of unknown primary	1	0	0	1/790 (0.13%)
Other tumor types	0	0	0	0/39,131 (0%)
Total	23	15	7	45/55,772 (0.08%)

^*^ Gliomas comprising anaplastic astrocytoma (*n* = 1), low‐grade glioma (*n* = 1), and glioblastoma (*n* = 4).

^†^ Lung carcinomas comprising adenocarcinoma (*n* = 4), small cell carcinoma (*n* = 2), and large cell neuroendocrine carcinoma (*n* = 1).

LOH of the *TP53* locus was observed in 30 of 40 (75%) evaluable cases (five cases were inconclusive due to insufficient heterozygous SNP markers in this genomic region). In 4 of the 10 tumors without LOH, a second *TP53* mutation was detected. Given that *TP53* is a prototypic tumor suppressor that conforms to the two‐hit model, the data support genetic inactivation of the remaining allele in a total of 34 of 40 (85%) cases, and generally consistent with c.375+5G variants being driver alterations. For the remaining 15%, the functional status of the remaining allele was unknown.

**Figure 1:**
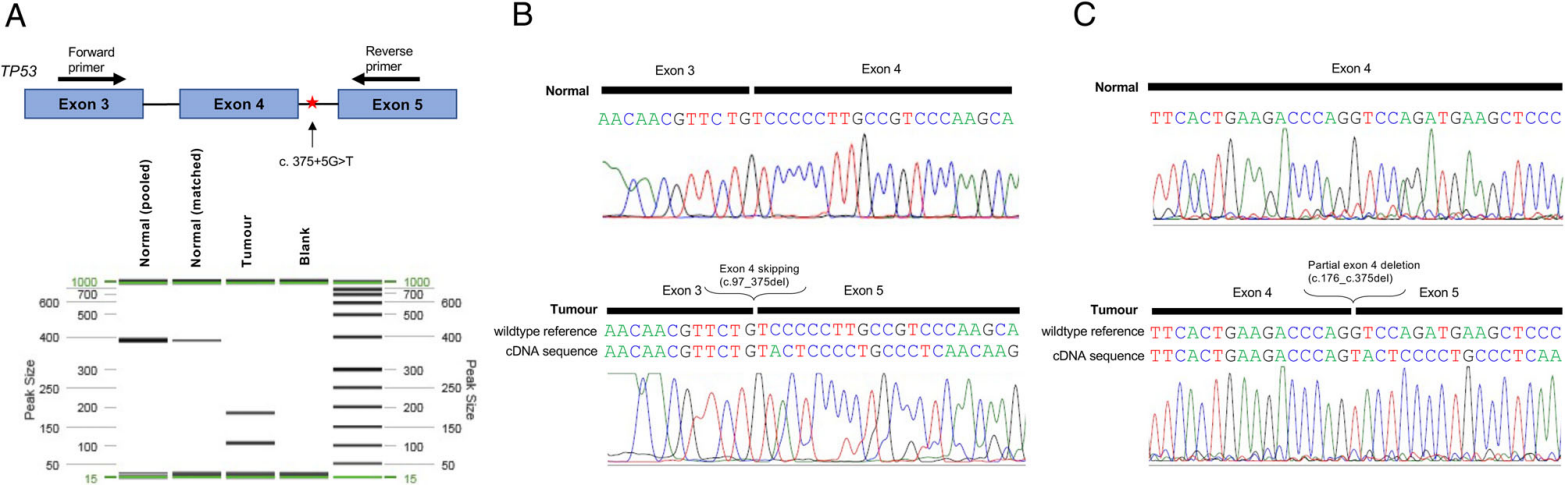
mRNA splicing analysis of the *TP53* c.375+5G>T variant. (A) RT‐PCR was performed using exon 3 and exon 5 primers (top), followed by capillary electrophoresis (bottom). (B and C) Direct sequencing of PCR products characterizing the aberrant transcripts.

To assess the impact of nucleotide substitution at c.375+5G on mRNA splicing, we analyzed *TP53* mRNA transcripts in a high‐grade serous ovarian carcinoma harboring the c.375+5G>T variant. Using exon 3 (forward) and exon 5 (reverse) primers flanking the intronic variant site, RT‐PCR was performed on mRNA extracted from tumor and normal controls. Capillary electrophoresis of RT‐PCR products revealed loss of the 382‐bp band corresponding to the wild‐type transcript, and the presence of two additional bands (103 and 182 bp), in tumor tissue (Figure 1A). Direct sequencing revealed corresponding transcripts with partial (r.176_375del) and complete (r.97_375del) exon 4 deletions, consistent with aberrant splicing of intron 4 (Figure 1B,C). The r.176_375del transcript introduces a premature stop codon, which, if translated, results in a truncated protein (p.Gly59Valfs*23), while exon 4 skipping is predicted to lead to an abnormal p53 protein product lacking part of the DNA‐binding domain (p.Ser33_Thr125del). Of note, the r.176_375del transcript was also detected when RT‐PCR was performed using an alternate exon 4 forward primer, and this transcript was previously reported in lung cancer, in association with a c.375+5G>A variant [10]. It is important to note that *TP53* splicing is extremely complex and our targeted approach may miss other aberrantly spliced transcripts not targeted by our analysis. Nevertheless, our results are consistent with an intact c.375+5G site being essential for generating the full‐length mRNA transcript, with nucleotide substitution being associated with truncated transcripts that may undergo nonsense‐mediated decay.

**Figure 2:**
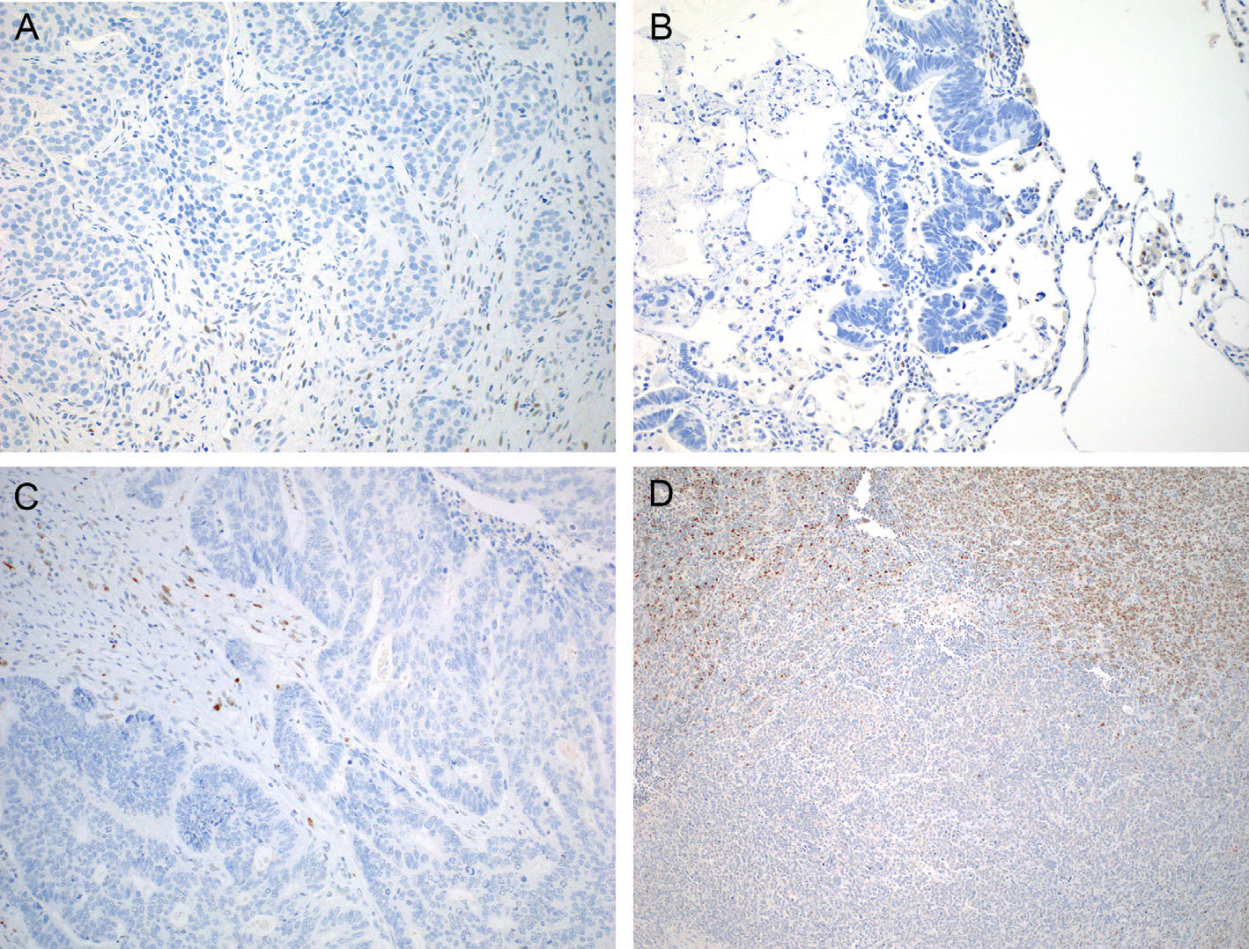
Immunohistochemical staining of p53 in representative tumors harboring *TP53* c.375+5G variants. While scattered normal stromal cells and lymphocytes in the stroma show weak nuclear staining, serving as an internal control, complete absence of p53 expression is seen in (A) invasive ductal carcinoma of breast (G>C), (B) metastatic colorectal adenocarcinoma (G>A), and (C) high‐grade serous ovarian carcinoma (G>T). In a case of (D) Merkel cell carcinoma with a subclonal G>A variant, there are distinct areas with loss of p53 expression adjacent to areas with intact expression.

Immunohistochemical staining for p53 was performed on tumor tissues from 14 cases (c.375+5G>T, *n* = 7; G>A, *n* = 4; G>C, *n* = 3). Complete loss of p53 protein expression throughout the tumor section was observed in 13 of these cases (93%; Figure 2A–C). The remaining case was a Merkel cell carcinoma demonstrating a heterogeneous staining pattern, with areas showing a wild‐type p53 expression pattern and distinct areas with complete absence of staining, suggestive of subclonal loss (Figure 2D). The variant allelic fraction for c.375+5G>A in this tumor was only 20% (compared to 81% for a coexisting *BCOR* mutation), and *TP53* LOH was present, which further support this interpretation. Overall, the immunohistochemistry results strongly support the notion that *TP53* c.375+5G somatic variants result in loss of p53 expression.

This study provides compelling evidence to support c.375+5G variants as deleterious in nature and observed across a range of tumor types. *In silico* analysis of all human splice sites across the genome has demonstrated guanine to be conserved at the +5 position in 77% of splice donor sites [11]. Our data show that, for the *TP53* gene, nucleotide substitutions at c.375+5G cause aberrant mRNA splicing, ultimately resulting in loss‐of‐function. Previous studies have demonstrated examples of intronic variants located some distance away from splice junctions to elicit aberrant mRNA splicing, likely through disruption of existing regulatory elements (e.g. splice enhancers or silencers) or creation of cryptic splice sites [10, 12]. For instance, a notable clinically relevant example is provided by *MET* exon 14 skipping in lung adenocarcinoma which can be caused by somatic mutations well into introns 13 and 14 of *MET* [13]. Furthermore, loss of p53 expression by immunohistochemistry should prompt consideration of an intronic splice‐altering mutation when a nonsense or frameshift mutation in the coding sequence is not identified. Diagnostically, identification of a *TP53* c.375+5G somatic mutation allows molecular confirmation of the histopathologic diagnosis when an exonic mutation is not present in a cancer type known to have a high prevalence of *TP53* genetic alterations, such as high‐grade serous ovarian carcinoma. Failure to identify *TP53* alterations may also have prognostic and therapeutic implications, as *TP53* inactivation is prognostically unfavorable across a wide spectrum of cancer types [1] and correctly establishing *TP53* status may soon become critical in clinical trials of MDM2 inhibitors. Finally, our findings highlight the opportunity provided by large‐scale, systematic clinical genomic profiling to identify novel or underappreciated driver alterations even in *TP53*, the most heavily sequenced cancer gene.

## Author contributions statement

MHC, ML and DM were involved in study design, data analysis, data interpretation, and writing of the manuscript. CY and VR carried out the experiments. NM, AZ and AM‐B analyzed the data. All authors had final approval of the submitted and published versions.
